# Assessment of Malawian Mothers’ Malaria Knowledge, Healthcare Preferences and Timeliness of Seeking Fever Treatments for Children Under Five

**DOI:** 10.3390/ijerph120100521

**Published:** 2015-01-09

**Authors:** Abayomi Samuel Oyekale

**Affiliations:** Department of Agricultural Economics and Extension, North-West University Mafikeng Campus, Mmabatho 2735, South Africa; E-Mail: asoyekale@gmail.com; Tel.: +27-787-144-271

**Keywords:** malaria, under-five children, healthcare preferences, Malawi

## Abstract

Malaria is one of the major public health problems in Malawi, contributing to the majority of morbidity and mortality among children under five. Ignorance of malaria symptoms results in delayed treatment, which often degenerates into fatal emergencies. This study analyzed the impact of maternal malaria knowledge on healthcare preferences and timeliness of treating children with reported fever. The Malaria Indicator Survey data for 2012, which were adequately weighted, were analyzed using multinomial logit and Poisson regression models. The results showed low maternal average years of formal education (3.52) and average mothers’ age was 27.97 years. Majority of the women (84.98%) associated fever with malaria, while 44.17% associated it with chilling. Also, 54.42% and 32.43% of the children were treated for fever on the same day and the following day that fever started, respectively. About 9.70% paid for fever treatment from their regular incomes, while 51.38% sought treatment from either public or private health centers. Multinomial Logit regression results showed that relative to using of other treatments, probabilities of selecting private hospitals and public health centers increased with age of the household heads, resident in urban areas, mothers’ years of education, number of days taken off for treatment, paying medical bills from regular, occasional and borrowed incomes, and knowledge of diarrhea and shivering as symptoms of malaria. In the Poisson regression results, timeliness of seeking treatment was significantly enhanced by knowledge of fever as malaria symptom, residence in northern and central regions of Malawi and use of income from sale of assets to pay medical bills (*p* < 0.10).However, delays in treating children was motivated by age of the household heads, number of days taken off to care for sick child and usage of regular, borrowed and other incomes to pay medical bills. (*p* < 0.05). It was concluded that efficiency of public sector in treating malaria holds significant prospects for fighting malaria in Malawi. However, adequate efforts should be channeled in enhancing the knowledge of women on malaria symptoms, among others.

## 1. Introduction

With estimated 3.4 billion people at risk of infection, malaria ranks among the major public health problems in the world today [1]. Available statistics have shown that countries in sub-Saharan Africa (SSA) bear the highest burdens of the disease’s associated morbidity and mortality [2,3,4]. It had been estimated that malaria is associated with more than 10% of disease burdens in Africa [5,6,7,8]. In many of these countries, tropical climate and poor sanitary conditions around residential homes often favor rapid multiplication of mosquitoes, which are the primary vectors of malaria parasite transmission [7,8]. In 2012, it was estimated that malaria resulted in 627,000 human deaths, although African children under the age of five years constituted the majority [1]. Socio-economic impact of malaria on the African economy is aggravated by poverty, which often prevents infected persons from seeking timely, qualitative and reliable treatments. Malaria also distorts national economic growth and development through sub-optimal allocation of productive resources, such as household members’ care giving time, lost working days by the sick person and imposition of substantial morbidity and mortality costs [1].

In Malawi, every segment of the population is directly exposed to the risk of malaria infection, although pregnant women and children exhibit significantly higher vulnerability [1,3]. Since 2000, Malawian government has not made significant progress in addressing malaria as one of the major public health problems the nation faces. This is the case, despite international assistances already received and collaborations among health professionals and stakeholders [9]. In 2010, 4 million cases of malaria and about 8000 malaria-associated deaths were reported in Malawi [10].

The quest for a world that is free of malaria had initiated international interventions and programs meant to enhance access to veritable malaria drugs, among others [10]. Specifically, some of these programs had addressed reduction in complications resulting from malaria among children under five through timely diagnosis and accessibility of effective home management [11,12,13]. Due to inequity in the distribution of health care services, substantial emphases had been placed on ensuring effectiveness of home management of malaria [12,13,14,15]. However, notable among the prerequisites for adequate home management of malaria is ability of mothers to properly diagnose malaria infection on a timely basis. This will ensure timeliness of chosen treatments. As primary caregivers, mothers have got a lot to offer in securing ultimate triumph over malaria in many African countries [14].

Furthermore, the need to reduce the socio-economic impacts of malaria has explored improvement in home treatment of fever among children with emphasis on some community-based initiatives like enhanced trainings for mothers, local drug vendors, and community health workers on adequate diagnosis, treatment and referrals [16,17]. Due to persistence of infection and high likelihood of fatality, early diagnosis and treatment of malaria among children under five can significantly reduce the level of associated mortality.

Dynamics of malaria infection and associated resistances to treatment over the past few decades had informed rapid changes in medical doctors’ treatment approaches [14,15]. Recently, artemisinin-based combination treatment (ACT) is reckoned to be the most effective treatment option to address drug resistances exhibited by malaria parasites [18].However, effectiveness of ACT depends on several factors, among which timely responsiveness to malaria symptoms through adequate seeking of treatments is essential. Over the years, medical scientists have also linked drug resistance of malaria parasite to unethical practices of traditional medicines and inability of patients to faithfully follow some prescribed orthodox medications.

Conceptually, the decision of households in response to illness can be influenced by several socio-economic and cultural factors [19,20]. Essentially, the choice of treatment can be directly related to affordability, availability and some sense of cultural beliefs on the perceived causes and severity of the illness [21]. Similarly, the choice made by households on where treatments are sought could determine treatment outcomes due to fundamental issues that are related to efficacy of prescribed drugs. Also, health policy makers can be properly guided to implement reforms for addressing malaria through research findings on the determinants of households’ health care choices [22,23,24]. Obviously, the informal health delivery system dominates the Malawian health care systems although formal treatments are often subsidized or rendered completely free of charge [25,26,27,28].

Oreagba *et al*. [29] conducted a study in Southwest Nigeria to determine the malaria knowledge and treatment choices of fever by mothers of children under the age of five. It was found that majority of the mothers had very low knowledge of malaria, while urban care givers reported higher use of health centers than their rural counterparts. In line with some previous studies [30,31,32,33,34], the majority of mothers indicated that chloroquine was the major drug used against malaria; there were misconceptions about right dosage, especially among residents in rural areas. In some other empirical studies, many factors had been found to influence health care seeking behavior of households. In many cases, individuals’ attitudes towards an ailment, households’ socio-economic status, community characteristics and cultural norms can have significant impacts on health seeking behaviors [25].

The response of mothers to child’s illness had been linked to Health Belief Model [35]. Timely detection and treatment of fever in children under five are linked to maternal socio-economic and demographic characteristics. This is associated with the fact that mothers play primary role in health issues affecting their children [29]. A study by Ajibade and Alao [35] found that early signs of fever that prompted mothers to take treatment initiatives were high body temperature and vomiting. It was further reported that attainment of formal education by mothers reduced the use of herbs significantly, while more than half of the respondents first consulted chemists for anti-malaria drugs.

Some previous studies had highlighted different mechanisms for addressing growing malaria problem in Malawi [3,6]. However, not much had been done on the effect of mothers’ malaria knowledge on timeliness of seeking treatments for fever among children under five and healthcare preferences. This is the gap that this study seeks to fill using the most recent data from Malawi’s Malaria Indicator Survey (MIS). This study therefore analyzed the maternal factors influencing decisions of timeliness and nature of treatment preferences for fever among under five children in Malawi. The first working hypothesis was that knowledge of malaria symptoms and danger signals possessed by mothers does not significantly influence health care preferences. The second hypothesis was that knowledge of malaria symptoms and danger signals possessed by mothers does not influence timeliness of seeking treatments for children under five that were sick with a fever. In the remaining parts of the paper, materials and methods, results, discussions, and conclusion had been presented.

## 2. Materials and Methods

### The Data and Sampling Methods

The study used the Malaria Indicator Survey (MIS) that was conducted in Malawi in 2012. The respondents were selected using two-stage sampling design. The sampling frame comprised of 140 clusters that were selected from 12,474 Enumeration Areas (EAs) identified in 2008 National Population and Housing Census. Out of the selected EAs, 96 were from rural areas, while 44 were from urban areas. At the second stage of sampling, involved selection of 25 households from each of the EAs using systematic sampling. In all, 3432 households were occupied out of the initially selected3500. Questionnaires were successfully administered to 3404 households and all the women between 15–49 years of age were interviewed. Also, 2955 women were eligible for women questionnaire out of which 2906 successfully completed the survey. The number of children under five was 2672, out of which 667 reported fever. Each household was appropriately weighted by the sampling weights presented in the data set in order to correct for sampling errors and for ensuring sample representativeness [36].Therefore, by integrating the sampling weight variable into various software for data analyses, all estimated parameters were properly weighted.

## 3. Analytical Models

### 3.1. Multinomial Logit Modeling of Healthcare Preferences 

The choice of where medical service for fever is sought for children under five is a discrete decision that is consistent with basic assumptions of discrete choice models. This model highlights the different sources from which health care services can be sought. Obviously, the model is adapted from the standard microeconomics theory of utility maximization in which utility depends on different attributes of health care choice *j* [37].The basis for using this model is based on inability to provide specific order for categorizing the relative importance of the choice of health care. It can be postulated that this choice is influenced by households’ demographic and socio-economic characteristics (*X*s). In the dataset used, the response variable (*y*) represented has the values of 1, 2 and 3 for use of public hospitals/clinics, private hospitals/clinics and other form of treatments like medicine shops, drug hawkers, *etc*. The model fits maximum-likelihood multinomial logit model, which is also known as polytomous logistic regression. In this case, a set of parameters denoted as β^1^, β^2^ and β^3^ which are corresponding to each of the health preference outcomes stated below:

(1) $$\text{Pr}\left(\text{y}=1\right)=\frac{e^{X\beta^{1}}}{e^{X\beta^{1}}+e^{X\beta^{2}}+e^{X\beta^{3}}}$$

(2) $$\text{Pr}\left(\text{y}=2\right)=\frac{e^{X{\text{β}}^{2}}}{e^{X{\text{β}}^{1}}+e^{X{\text{β}}^{2}}+e^{X{\text{β}}^{3}}}$$

(3) $$\text{Pr}\left(\text{y}=3\right)=\frac{e^{X{\text{β}}^{3}}}{e^{X{\text{β}}^{1}}+e^{X{\text{β}}^{2}}+e^{X{\text{β}}^{3}}}$$

This model is unidentified due to possibility of having more than one solution to β^1^, β^2^ and β^3^ that gives the same probabilities for *y* = 1, *y* = 2 and *y* = 3. In order to address this problem, one of the parameters should be arbitrarily set to zero, which now forms the reference group. In this study, response variable 1 (use of other form of treatments) was set as the reference group and relative risk ratios were computed for each parameter. For instance: the relative probability of *y* = 2 to the base is expressed as:

(4) $$\frac{Pr(\text{y}=2)}{\text{Pr}(\text{y}=1)}=e^{X{\text{β}}^{2}}$$

The models were estimated with STATA 13 software and sampling weight was selected before carrying out the econometric analyses. The parameters to be estimated (β*_i_*) are presented, while *X_k_* represents the included explanatory variables. Multicollinearity among the variables was tested with variance inflation factor (VIF). High levels of tolerance computed for all the variables signal absence of serious multicollinearity. The included explanatory variables were coded as follows: age of household head (years), danger signs of malaria: high fever (*yes* = 1, 0 otherwise), type of place of residence (*urban* = 1, 0 otherwise), knows main malaria symptoms: Refuse to eat or drink (*yes* = 1, 0 otherwise), danger signs of malaria: restless (*yes* = 1, 0 otherwise), knows main malaria symptoms: diarrhea (*yes* = 1, 0 otherwise), Northern region (*yes* = 1, 0 otherwise), Central region (*yes* = 1, 0 otherwise), highest year of education, danger signs of malaria: fainting (*yes* = 1, 0 otherwise), danger signs of malaria: any fever, (*yes* = 1, 0 otherwise), danger signs of malaria: stiff neck (*yes* = 1, 0 otherwise), danger signs of malaria: chills/shivering, (*yes* = 1, 0 otherwise), danger signs of malaria: unable to eat (*yes* = 1, 0 otherwise), danger signs of malaria: diarrhea (*yes* = 1, 0 otherwise), number of days taken off (*yes* = 1, 0 otherwise), knows main malaria symptoms: chills (*yes* = 1, 0 otherwise), knows main malaria symptoms: headache (*yes* = 1, 0 otherwise), knows main malaria symptoms: dizziness (*yes* = 1, 0 otherwise), knows main malaria symptoms: body ache or joint pain (*yes* = 1, 0 otherwise), source of payment for treatment (income) (*yes* = 1, 0 otherwise), source of payment for treatment (occasional income) (*yes* = 1, 0 otherwise), source of payment for treatment (borrowed) (*yes* = 1, 0 otherwise), source of payment for treatment (sale of assets) (*yes* = 1, 0 otherwise), and source of payment for treatment (other) (*yes* = 1, 0 otherwise).

### 3.2. Timeliness of Treatment Modeling With Poisson Regression

Because the dependent variable was a count of the number of days before treatments were sought, parameter estimation with Poisson regression was the most appropriate. Therefore, a random integer variable *T* with values of 0, 1, 2, 3, 4, *n* was modeled with a Poisson distribution with parameter μ [37]. The probability distribution of such variable was expressed as:

(5) $$\text{Pr}\left\{T=t\right\}=\frac{e^{-\mu }\mu^{t}}{t!}\text{for}\mu \;>\;0$$

The model is specified as:

(6) $$lnT_{i}=\text{α}+{\text{β}}_{j}\overset{k}{\underset{j=1}{\sum }}X_{k}$$

**Figure 1:**
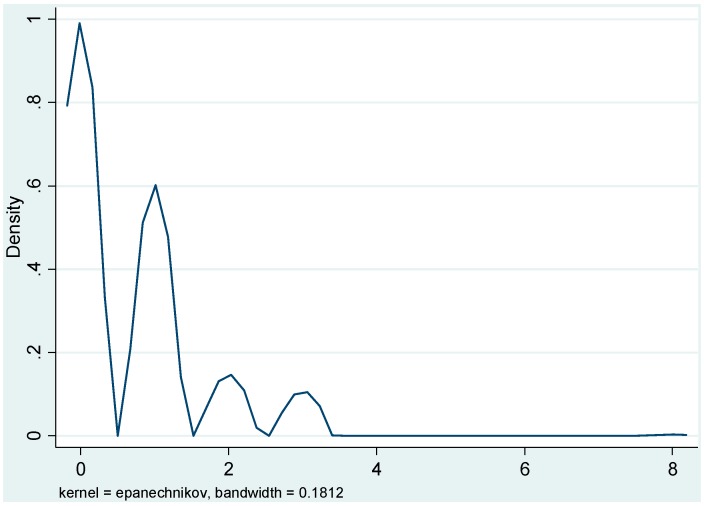
Kernel density graph of the days mothers waited before treating children infected with malaria.

Given assumption of heteroscedasticity, Maximum Likelihood Estimation (MLE) method is always used to estimate parameters in Poisson regression. However, the model’s goodness of fit was assessed from statistical significance of deviance statistics. If this shows statistical significance (*p* < 0.05), it implies that Poisson distribution assumption had been violated. Therefore a better model, such as negative binomial regression model should be used. The kernel density graph of the dependent variable—mean was 0.6701 and the standard deviation was 0.9005—is presented in Figure 1. The graph shows confirms that the distribution was positively skewed emphasizing non-suitability of Ordinary Least Square (OLS) regression.

## 4. Results

### 4.1. Socio-Economic Profiles, Malaria Knowledge and Fever Treatment Preferences

Table 1 presents the descriptive statistics of children’s and mothers’ selected socio-economic characteristics. It reveals that average age of the children was 28.15 months with standard deviation of 15.57. Also, about 49% of the children were males. Average age of the mothers was 27.97 years, while their average years of formal education was 3.52 years. About 79% of the household heads were male and average number of children under five was 1.63. The table further shows that only about 11% of the children were resident in urban areas and majority were from northern and central regions.

The questionnaire probed into the knowledge of the mothers on symptoms of malaria. The results in Table 2 showed that majority of the women (84.98%) associated fever with malaria, while 44.17% associated it with chilling. Nausea or vomiting was associated with malaria by 40.73% of the women, while 23.78% associated it with body ache or joint pains. High fever and convulsion were associated with danger signals of malaria in 22.44% and 44.52% of the children.

**Table 1:** Descriptive statistics of the mothers’ and children’s socio-economic characteristics.

Variables	Mean	Std. Deviation	Minimum	Maximum
Number of under five children	1.63	0.7063	0	4
Sex of child	0.49	0.4998	0	1
Child's age in months	28.15	15.5719	0	59
Age of household head	36.26	15.0545	16	98
Urban residence	0.11	0.3074	0	1
North Region	0.11	0.3098	0	1
Central Region	0.44	0.4968	0	1
South Region	0.45	0.4974	0	1
Highest mothers’ years education	3.52	2.7047	0	8
Mothers’ age	27.97	6.5819	15	48
Male household headship	0.79	0.4071	0	1

Note: Results were weighted.

**Table 2:** Percentage distribution of mothers’ malaria knowledge.

Variables	Percentage
*Knowledge of Malaria Danger Signals*	
Danger signs of malaria: Seizure/convulsions	44.52
Danger signs of malaria: Fainting	17.37
Danger signs of malaria: Any fever	7.41
Danger signs of malaria: High fever	22.44
Danger signs of malaria: Stiff neck	3.63
Danger signs of malaria: Feeling weak	11.38
Danger signs of malaria: Not active	0.78
Danger signs of malaria: Chills/shivering	7.90
Danger signs of malaria: Unable to eat	2.39
Danger signs of malaria: Vomiting	9.94
Danger signs of malaria: Crying all the time	1.40
Danger signs of malaria: Restless	2.36
Danger signs of malaria: Diarrhea	4.29
Danger signs of malaria: Other	4.92
Danger signs of malaria: Don’t know	11.79
*Knowledge of Malaria Symptoms*	
Knows main malaria symptoms: fever	84.98
Knows main malaria symptoms: chills	44.17
Knows main malaria symptoms: headache	17.22
Knows main malaria symptoms: Nausea/vomiting	40.73
Knows main malaria symptoms: Diarrhea	13.29
Knows main malaria symptoms: Dizziness	1.85
Knows main malaria symptoms: Loss of appetite	3.90
Knows main malaria symptoms: Body ache or joint pain	23.78
Knows main malaria symptoms: Pale eyes	2.80
Knows main malaria symptoms: Feeling weak	6.35
Knows main malaria symptoms: other	2.42

Note: Results were weighted.

Table 3 shows the distribution of the days sick, days before seeking treatment and health care preferences in Malawi. In the database, there were a total of 2672 under five children, of which 667 reported fever within previous 14 days of the survey. This implies that about one out of four under five children reported fever. However, table 3 shows that 20.20% and 23.31% of the children had malaria three and seven days prior to the survey, respectively. In the combined data, 52.92% and 32.98% of the children were treated for fever on the same day and the following day that sickness started. However, treatment of fever was sought by majority of the children (54.42%) on the same day fever was noticed, while 32.43% sought treatment one day after. Also, 46.89% sought treatments from public hospitals and health centers and only 4.59% of the children were treated in private hospitals. The highest proportion of the children (48.52%) was treated in other facilities, including self medication and traditional healers.

**Table 3:** Distributions of fever sick days, days before treatment and health care preferences.

Variables	Frequency	Percentage
*Days ago had malaria*		
0	9	1.41
1	33	4.64
2	105	14.02
3	143	20.20
4	52	7.66
5	48	8.02
6	26	3.98
7	152	23.31
8	25	3.85
9	13	2.25
10	32	5.30
11	1	0.23
12	9	1.37
13	6	0.68
14	22	3.07
*Days after fever sought treatments*		
0	353	54.42
1	220	32.43
2	54	6.81
3	40	6.16
8	1	0.18
*Healthcare Preferences*		
Public hospital/health centers	317	46.89
Private hospital/health centers	31	4.59
Other sources	328	48.52

Note: Results were weighted.

Table 4 shows that 9.70% of the children’s parents paid for fever treatment from their regular incomes, while 4.21% paid from the proceeds from sale of assets. Also, 4.79% of the children were treated by occasional incomes.

**Table 4:** Percentage distribution of sources incomes used for malaria treatments.

Variables	Percentage
*Sources of Malaria Treatment Fees*	
Regular Income	9.70
Occasional income	4.79
Borrowed income	1.04
Sale of assets	4.21
Other incomes	1.40

Note: Results were weighted.

### 4.2. Factors Influencing Health Care Preferences

Table 5 shows the results of multinomial logit regression with the relative risk ratios. The reference group is “use of other forms of treatment”. The Wald chi-square value was statistically significant (*p* < 0.01). This implies that the coefficients of the parameters estimated for the included variables were not all statistically equal to zero. It also indicates that the model produced a good fit for the data. High tolerance levels of the explanatory variables give some indications of absence of multicollinearity. Since some of the variables that were included to capture knowledge of malaria showed statistical significance, the first null hypothesis is hereby rejected.

The parameters of age of household head were statistically significant in the public health center (*p* < 0.05) and private health center (*p* < 0.01) models. Increase in age increased the probabilities of selecting public and private health centers relative to other forms of treatments. Also, if the age of household heads increased by one year, the relative risk ratios of selecting public and private health centers would be higher by 1.0161 and 1.0589, respectively. Residence in urban areas also significantly increased probabilities of treating children infected with fever in public and private health centers (*p* < 0.05). However, the relative risk ratios of selecting public and private health centers by urban residents increased by 1.9217 and 9.2108, respectively.

Also, relative to those who were in southern region, resident in northern region significantly reduced probability of selecting public health centers (*p* < 0.05) and private health centers (*p* < 0.01). Also, resident in central region significantly reduced the probability of using public health centers (*p* < 0.05). The risk of using public health centers increased by 0.6004, relative to those in rural areas holding other variables constant. As the years of mothers’ education increased, probabilities of choosing public and public health centers increased significantly. If the years of maternal education increased by one year, the relative risk ratio of selecting public and private health centers increased by 1.0654 (*p* < 0.10) and 1.2712 (*p* < 0.05), respectively. The number days taken off by mothers from work to take care of fever infected children in public health centers showed statistical significance (*p* < 0.05). This implies that probabilities of using public centers relative to other health facilities increased significantly with the number of days taken off by women to care for the children. Relative risk ratio of selecting public health centers was higher by 1.4683 relative to using other health facilities as the number of days taken off by mothers to take care of infected children increased.

**Table 5:** Multinomial logit regression results of factors explaining healthcare preferences.

Variables	Public Health Centers			Private Health Centers			Tolerance
	Coefficient	RRR	*z*-stat	Coefficient	RRR	*z*-stat	
*Demographic variables*							
Age of household head	0.0160	1.0161	2.35	0.0573	1.0589	2.97	0.9708
Type of place of residence	0.6532	1.9217	2.33	2.2204	9.2108	3.75	0.8444
Northern region	−0.7258	0.4839	−2.35	−29.3764	0.0000	−24.12	0.7659
Central region	−0.5102	0.6004	−2.27	−0.5992	0.5492	−0.81	0.7014
Highest year of education	0.0633	1.0654	1.78	0.2400	1.2712	2.02	0.9252
Number of days taken off	0.3841	1.4683	2.42	0.3112	1.3651	0.86	0.8089
*Danger signals of malaria*							
Danger signs of malaria: High fever	0.2237	1.2507	0.95	−0.3675	0.6925	−0.66	0.8811
Danger signs of malaria: Restless	−1.0087	0.3647	−1.58	0.6106	1.8416	0.58	0.9211
Danger signs of malaria: Fainting	0.2090	1.2324	0.80	0.3693	1.4468	0.53	0.7909
Danger signs of malaria: Any fever	−0.0457	0.9553	−0.14	−0.4657	0.6277	−0.53	0.9111
Danger signs of malaria: Stiff neck	−1.1541	0.3153	−2.08	−1.0175	0.3615	−0.93	0.9471
Danger signs of malaria: Chills/shivering	0.4260	1.5312	1.02	1.5705	4.8091	1.87	0.9033
Danger signs of malaria: Unable to eat	0.4013	1.4938	0.76	−1.7407	0.1754	−1.60	0.9062
Danger signs of malaria: Diarrhea	0.3035	1.3546	0.62	0.1678	1.1827	0.22	0.9165
Knows main malaria symptoms: Diarrhea	0.0836	1.0872	0.30	2.0943	8.1200	2.66	0.9002
Knows main malaria symptoms: chills	−0.3434	0.7094	−1.67	−0.5909	0.5538	−0.93	0.8442
Knows main malaria symptoms: headache	0.6356	1.8881	2.43	−0.3394	0.7122	−0.46	0.8563
Knows main malaria symptoms: Dizziness	0.7073	2.0286	1.02	−26.2484	0.0000	−23.10	0.9235
Knows main malaria symptoms: Body ache or joint pain	0.3103	1.3638	1.31	−0.0231	0.9772	−0.03	0.9067
Source of payment for treatment (Income)	1.3009	3.6726	3.56	4.1525	63.5925	5.65	0.8444
Source of payment for treatment (Occasional income)	0.0797	1.0830	0.17	3.7961	44.5260	4.11	0.9165
Source of payment for treatment (Borrowed)	2.5953	13.4006	2.24	2.8936	18.0573	1.80	0.9408
Source of payment for treatment (Sale of assets)	−1.7047	0.1818	−1.60	1.8172	6.1543	1.35	0.8561
Source of payment for treatment (Other)	−1.8972	0.1500	−2.76	2.2981	9.9551	1.91	0.9708
Constant	−0.8924	0.3346	−2.67	−7.9550	0.0004	−5.54	
Log likelihood	−239.8497						
Number of observations	667						
Wald Chi Square (50)	4969.90 ***						
Pseudo R square	0.1719						
Knows main malaria symptoms: Diarrhea	0.0836	1.0872	0.30	2.0943	8.1200	2.66	0.9002
Knows main malaria symptoms: chills	−0.3434	0.7094	−1.67	−0.5909	0.5538	−0.93	0.8442
Knows main malaria symptoms: headache	0.6356	1.8881	2.43	−0.3394	0.7122	−0.46	0.8563

*** Results were weighted.

Furthermore, holding other variables constant, children whose parents paid for fever treatment from their regular incomes had significantly higher probability of being treated in public and private hospitals (*p* < 0.01). Also, for those children whose parents paid the fees for fever treatment from regular income, the relative risk ratio for selecting public and private health centers was significantly higher (*p* < 0.01) by 3.6726 and 63.5925, respectively, when compared to those treated in other facilities. The results further indicated that relative to those children that were treated in other facilities, children whose parents paid for fever treatments from occasional incomes had significantly higher probability of being treated in private hospitals (*p* < 0.01). Holding other variables constant, payment from occasional income significantly increased the relative risk ratio for using private health centers by 44.5260 (*p* < 0.01).

Borrowing money to pay bills significantly increased the probabilities of seeking fever treatments from public and private health centers (*p* < 0.05). The relative risk ratios for those children whose parents borrowed money to pay bills in public and private health centers were significantly higher by 13.4006 (*p* < 0.05) and 18.0573 (*p* < 0.10), respectively, when compared to those that were treated in other facilities.

Also, children whose parents paid for fever treatment from other incomes were with significantly higher probability of being treated in private health centers (*p* < 0.10). The relative risk ratio for selecting private health centers was higher by 9.9551 for this category of children. Similarly, children whose fever treatment bills were paid from incomes realized from other sources had significantly lower probability of being treated in public health centers (*p* < 0.01). Compared to using other form of treatments, the relative risk ratio for selecting public health centers was significantly lower by 0.1500 (*p* < 0.01).

Furthermore, knowledge of malaria significantly influenced health care preferences. Precisely, the knowledge of refusal to eat as symptoms of malaria significantly reduced the probability of selecting private centers for treatment (*p* < 0.01). The relative risk ratio for using private health centers was also reduced among those that indicated refusal to eat as symptom of malaria. Those mothers that indicated diarrhea as malaria symptom had significantly higher probability (*p* < 0.01) of selecting private health center for treating children infected with fever. Also, holding other variables constant, the relative risk ratio of selecting private health centers for treating fever in children was higher by 8.1200 for the children whose mothers knew diarrhea as symptom of malaria.

The mothers that indicated chills as symptom of malaria had significantly lower probability of selecting public health centers (*p* < 0.10). The relative risk ratio for selecting public health centers among those that indicated chills as symptom of malaria was higher by 0.7094 when compared to those treated in other facilities. In addition, knowledge of headache by the mothers as symptom of malaria significantly increased probability of selecting public health centers (*p* < 0.05). The relative risk ratio for selecting public health centers among those that indicated headache as symptom of malaria was higher by 1.8881 when compared to those treated in other facilities. The children whose mothers indicated dizziness as symptom of malaria had significantly lower probability of selecting private health centers (*p* < 0.01). The relative risk ratio for selecting private health centers among those that indicated dizziness as symptom of malaria was higher by very small value when compared to those treated in other facilities.

The children whose mothers indicated stiff neck as danger signal of malaria had significantly lower probability of selecting public health centers (*p* < 0.05). The relative risk ratio for selecting private health centers among those that indicated stiff neck as symptom of malaria was higher by 0.3153 when compared to those treated in other facilities. The children whose mothers indicated shivering as danger signal of malaria had significantly higher probability of selecting private health centers (*p* < 0.10). The relative risk ratio for selecting private health centers among those that indicated shivering as danger signal of malaria was higher by 4.8091 when compared to those treated in other facilities

### 4.3. Factors Influencing Timeliness of Fever Treatment

Table 6 contains the results of Negative Binomial regression. These results were generated after the Poisson model was found to be inappropriate based on statistical significance of the Deviance goodness of fit and Pearson goodness of fit statistics (*p* < 0.01). The computed log-pseudo likelihood function is −7.488e + 8. However, statistical significance of the Wald Chi square value (*p* < 0.01) implies that the estimated parameters were not jointly equal to zero. Some of the variables that captured knowledge of malaria showed statistical significance (*p* < 0.10). This implies that the second null hypothesis should be rejected.

**Table 6:** Negative Binomial regression results of factors influencing treatment timeliness.

Variables	Coefficients	Robust Std. Error	*Z* Statistics	Tolerance
*Demographic factors*				
Age of household head	0.0118	0.0029	4.04	0.9708
Type of place of residence	0.1334	0.1215	1.10	0.8444
Northern region	−0.3975	0.1689	−2.35	0.7659
Central region	−0.3378	0.1198	−2.82	0.7014
Highest year of education	0.0304	0.0199	1.53	0.9252
Number of days taken off	0.1358	0.0610	2.23	0.8089
*Danger signals of malaria*				
Danger signs of malaria: High fever	0.1359	0.1267	1.07	0.8811
Danger signs of malaria: Restless	−0.6570	0.4253	−1.54	0.9211
Danger signs of malaria: Fainting	−0.2343	0.1469	−1.59	0.7909
Danger signs of malaria: Any fever	−0.7472	0.2084	−3.59	0.9111
Danger signs of malaria: Stiff neck	−0.4624	0.4182	−1.11	0.9471
Danger signs of malaria: Chills/shivering	0.2162	0.1853	1.17	0.9033
Danger signs of malaria: Unable to eat	−0.3452	0.3243	−1.06	0.9062
Danger signs of malaria: Diarrhea	−0.3547	0.2391	−1.48	0.9165
*Knowledge of malaria symptoms*				
Knows main malaria symptoms: Refuse to eat or drink	−0.9128	0.6461	−1.41	0.8902
Knows main malaria symptoms: Diarrhea	0.1395	0.1478	0.94	0.9002
Knows main malaria symptoms: chills	−0.1443	0.1113	−1.30	0.8442
Knows main malaria symptoms: headache	0.1969	0.1380	1.43	0.8563
Knows main malaria symptoms: Dizziness	0.4323	0.2609	1.66	0.9235
Knows main malaria symptoms: Body ache or joint pain	0.1287	0.1248	1.03	0.9067
Source of payment for treatment (Income)	0.8694	0.1336	6.51	0.8444
Source of payment for treatment (Occasional income)	0.8170	0.2494	3.28	0.9165
Source of payment for treatment (Borrowed)	0.3054	0.4395	0.69	0.9408
Source of payment for treatment (Sale of assets)	0.9908	0.2335	4.24	0.8561
Source of payment for treatment (Other)	0.9650	0.3176	3.04	0.9708
Constant	−1.0611	0.1694	−6.26	
lnalpha	−3.4010	2.7538		
Alpha	0.0333	0.0918		
Number of obs = 667				
Log pseudo likelihood = −7.488e + 08				
Wald Chi Square (25) = 190.96 ***				

*** Results were weighted.

Holding other variables constant, the children from northern region of Malawi had a significantly lower log of days before being treated for fever, by 0.3975 (*p* < 0.05). Also, if other variables are held constant, the children from central region of Malawi had a significantly lower log of days before being treated for fever by 0.3378 (*p* < 0.01).However, as the age of household heads increased by one year (holding other variables constant), log of days mothers waited before seeking treatments for children with fever symptoms significantly increased by 0.0118 (*p* < 0.01).

Holding other variables constant, the log of number of days waited before treatment for the children whose parents paid with regular income was significantly higher by 0.8694 (*p* < 0.01). Also, the children whose parents paid treatment fees through occasional income had a significantly higher log of number days waited before treatment by 0.8170 (*p* < 0.01). In addition, holding other variables constant, the children that got their fever treatment costs paid through parents’ sale of assets and other sources of income had a being significantly higher log of days waited before treatment by 0.9908 and 0.9650, respectively (*p* < 0.01).

The parameter of knowledge of any fever as a danger signal of malaria is with negative sign. It also implies that the log of number of days waited before treatment for the children whose parents knew any fever as danger signal of malaria was significantly lower by 0.7472 (*p* < 0.01). Also, the log of number of days waited before treatment for the children whose parents knew dizziness as symptom of malaria was significantly higher by 0.4323 (*p* < 0.10). As the number of days taken off from work by mothers to take care of children infected by fever increased by one day, the log of number of days waited before treatment significantly increased by 0.1358 (*p* < 0.05).

## 5. Discussions

Average year of formal education by the mothers was 3.52 years, which implies very low educational attainments. Low attainment of formal education could also influence knowledge of malaria symptoms and use of preventive and curative practices [38]. Several studies have emphasized the importance of maternal education for child health [39,40]. A framework presented by Mosley and Chen [41] directly linked maternal education to children’s health through enhancement of women’s socioeconomic status. It was noted that education would influence women’s fertility factors, child’s feeding practices, and the healthcare preferences [40].

World Health Organization (WHO) noted that early malaria symptoms could include fever, headache, chills and vomiting [42]. The results indicated that the knowledge of the women on critical signs of malaria was very low. This is obviously a reflection of the, on average, low educational attainments among the mothers as previously reported by Sharma [43] for some women in India. With 84.98% associating fever with malaria, the knowledge of women on malaria’s critical symptoms was very high. In a similar study in Kenya, majority of women associated headache (70%) and fever (68.8%) with malaria [44]. With 40.73% associating malaria infection in children with vomiting, the result was quite different from what was found in Kenya where 0.5% was computed [43]. The results are also bringing to fore the essence of health education interventions for reducing the impact of malaria of children. In some related studies [45], interventions to enhance knowledge of children’s primary caregivers enhanced right diagnosis of malaria and administration of right doses of drugs.

Fever was reported by about 25% of children under five in the survey. Some previous studies had reported high rate of fever among children under five from Malawi [46,47]. In purely rural settings, malaria prevalence had been found to be as high as 45.6% [47]. Also, in line with recommendation of the World Health Organization (WHO), majority of the children were treated within 24 hours of showing malaria symptoms [42]. This was as a result of high level of awareness of malaria and understanding of its possible consequences on households’ welfare. Timely treatment of malaria had been noted as a panacea for reducing transmission of malaria parasites from person to person and reduction of emergency from severity of infection [22,42].

Although majority of the children were treated outside public and private hospitals, appreciable number were treated in government’s public health centers. In Malawi, incentives for using public hospitals’ had been provided by free treatment of malaria [27]. However, it had been noted that growing poverty among Malawian households is a major bottleneck influencing their healthcare seeking behavior. Though the government provides free malaria treatment in public hospitals, transportation cost and required traveling time may also constitute serious constraints among poverty stricken households [27]. Therefore, several issues that are related to socio-economic development are critical determinants of healthcare preferences in Malawi [48].

Furthermore, it had been emphasized that majority of malaria treatments in Malawi occur outside the formal sector although free treatments are given in public hospitals/health centers [26,27,28]. Some households often seek formal treatment of malaria after the failure of self-administered treatments [25]. Also, it had been noted that inability to access adequate malaria treatments in public health centers that offer free treatment limit access to effective treatments by the poor [49].

In the parametric results, utilization of public and private health services increased with the age of household heads, specifically, age influences healthcare seeking behavior through previous experiences and possibility of having enough income for medical bills. In a previous study, age was found to be an insignificant determinant of healthcare seeking behavior [50] while it showed statistical significance in some others [23]. The age of the household head also motivated delay in treating the children that were infected with fever. This may have resulted from likelihood of doubt of what specific health challenges the child may be having. It is also important that experiences of older household heads can reestablish the nature of infection suffered by children [24,51,52].

Resident of urban areas have significantly higher probabilities of using public and private health centers. Utilization of more orthodox medical services by urban resident could have been motivated by concentration of these services in urban areas. Many previous studies have noted the differentials in health care services between rural and urban areas [53,54,55,56]. However, understanding the factors influencing access to orthodox medical services by urban and rural dwellers calls for critical evaluation of issues, such as income, educational status and proximity [57,58,59]. This assertion underscores the significant influences that some education and sources of income variables have on choice of health services and timeliness of seeking treatments. Poverty had been identified as a major determinant of health status, which obviously has a lot to do with the quality of treatment and its timeliness [50,60,61,62]. Maternal education also influences the income level and health care choice and consumption. It is important to note that decisions to obtain health insurance and use private orthodox medical services can be influenced by educational attainment of the mother.

## 6. Conclusions

Malaria is a major public health challenge in many developing countries. In Malawi, it accounts for the majority of morbidity and mortality among children under five. This paper provides a clear evaluation of maternal knowledge on basic malaria symptoms and its impacts on the choice of healthcare services and timeliness of seeking treatment for children under five with reported fever. The Malawian government had taken several initiatives to address malaria as a limiting factor for households’ socio-economic development; several environmental and socio-economic problems constitute barriers to achievement of any significant impacts. In this study, it was emphasized that understanding the factors influencing timelines of seeking treatment for children with malaria infection is prerequisite for reducing emergencies resulting from delays in malaria treatments and associated mortality. The findings have highlighted low educational attainments and knowledge of malaria among the women. This raises the need for enhancing the knowledge of women on necessary health issues through counseling while seeking health care services. Majority of the children were treated freely but usage of orthodox health care services was low. This raises concerns on quality of services rendered in those free health centers and waiting time. This obviously calls for direct evaluation of healthcare service facilities in Malawi in order to ensure adequate coverage and reduction in waiting time. The preferences made on where children received malaria treatment were highly influenced by the source of incomes for paying medical bills. However, many poor households would seek free malaria treatment from public hospitals, but that should not be at the detriment of quality and efficiency. The findings further emphasized the need for the Malawian government to enhance welfare of people through capacity development and skill building activities.
